# The Effect of Using a Camouflaged Dental Syringe on Children’s Anxiety and Behavioral Pain

**DOI:** 10.7759/cureus.50023

**Published:** 2023-12-06

**Authors:** Sara M Bagher, Osama M Felemban, Ghufran A Alsabbagh, Noura A Aljuaid

**Affiliations:** 1 Pediatric Dentistry, Faculty of Dentistry, King Abdulaziz University, Jeddah, SAU; 2 Pediatric Dentistry, King Abdulaziz University Dental Hospital, Jeddah, SAU; 3 Primary Health Care, Ministry of Health, Albaha, SAU

**Keywords:** camouflaged dental syringe, pediatric dentistry, local anesthesia, dental pain, dental anxiety

## Abstract

Background: Seeing a dental syringe can be terrifying, especially for young children, and hiding it during local anesthesia (LA) administration can sometimes be challenging for the pediatric dentist.

Objective: To assess the effect of a camouflaged dental syringe on children’s anxiety and behavioral pain in comparison to the traditional dental syringe during local anesthesia administration in pediatric patients.

Materials and methods: This randomized clinical trial included cooperative and healthy 6- to 10-year-old children scheduled for non-urgent dental treatment that required buccal infiltration anesthesia (BIA) in the maxillary arch. The subjects were randomized into either the test or the control groups. In the test group, subjects received BIA using the camouflaged dental syringe. Subjects in the control group received the BIA using a traditional dental syringe. A single-trained dentist administered all the anesthesia. Heart rate (HR) was monitored at three different time points (before, during, and after) the BIA administration. Subjects’ anxiety and behavioral pain were measured using Venham’s Anxiety Rating Scale (VARS) and the Face, Leg, Activity, Cry, and Consolability (FLACC) scale, respectively, by two trained and calibrated investigators.

Results: A total of 60 subjects with a mean age of 8.3 ±1.3 years were included. The scores of the VARS in the subjects in the camouflaged group were somewhat lower than the subjects in the traditional group, but the observed difference did not reach statistical significance (*P*=0.113). However, subjects in the camouflaged group showed significantly lower FLACC scores compared to the traditional group (*P*=0.034).

Conclusion: The utilization of a camouflaged dental syringe is effective in improving children’s behavior during local anesthesia administration; therefore, it is recommended as an alternative to using the traditional syringe.

## Introduction

Local anesthesia (LA) is defined as a loss of sensation in a localized area of the body by inhibiting the conduction process in peripheral nerves or depressing the excitation in the nerve endings [1]. In dentistry, the administration of LA is challenging, as needle phobia is considered one of the leading causes of dental fear and anxiety, especially among young children [2,3].

Building a good and trusting relationship between the dentist and a child patient is a crucial factor for maintaining the child’s cooperation during treatment [4]. Once trust is gained between the child patient and their dentist, a positive feeling toward dental visits is formed and stored in the child’s memory. Keeping tools and instruments out of sight -including the dental syringe - that might be scary-looking from a child’s perspective is considered a critical factor in minimizing anxiety and fostering trust between child patients and their dentist [5].

Hiding the dental syringe from a child's sight during LA administration can be challenging. Distracting the child by camouflaging the dental syringe can be useful; therefore, a newly developed autoclavable, colorful, and playful alligator-shaped syringe sleeve to cover and camouflage the threatening metal dental syringe during LA administration can be an effective distraction tool to alleviate dental anxiety [6]. Utilizing the sleeves allows the dentist to administer the anesthesia while eliminating the visual input of the metal syringe and needle.

A limited number of studies have evaluated the influence of the alligator-shaped sleeves on the anxiety of children and their behavior during the LA administration; improvements in behavioral pain and reduced anxiety associated with LA administration in pediatric patients with no previous experience with dental anesthesia have been reported [7,8].

Therefore, our study aimed to evaluate the effect of a camouflaged dental syringe in comparison to the traditional dental syringe during local anesthesia administration on the anxiety level and behavioral pain among 6- to 10-year-old cooperatively healthy children with previous dental anesthesia experience. We hypothesized (the alternative hypothesis) that using a camouflaged dental syringe would decrease anxiety and improve behavior among this population.

## Materials and methods

This randomized clinical trial was conducted at the pediatric dentistry clinics at the King Abdulaziz University Faculty of Dentistry (KAUFD). Ethical approval was obtained from the Research Ethical Committee at King Abdulaziz University, Faculty of Dentistry (053-02-19). The study was reported according to the protocol established by the Consolidated Standards of Reporting Trials (CONSORT) statement, with a checklist used for randomized clinical trials [9]. The study protocol was registered in the clinicaltrials.gov database with the identifier NCT06116994.

The dental records of all the children scheduled for non-urgent dental treatment between January and May 2023 at the Pediatric Dentistry Department clinics were reviewed to identify potentially eligible subjects. Inclusion criteria included healthy and cooperative 6- to 10-year-old children with no known allergy and/or sensitivity to LA who are scheduled for non-urgent dental treatment that requires buccal infiltration anesthesia (BIA) of at least one maxillary molar. The cooperation level of the children was determined based on the child’s behavior recorded in the dental file from their previous dental visits. Only children who were reported to be positive or absolutely positive according to Frankl’s behavior rating scale were considered eligible [10]. Children reported to be uncooperative during their previous dental visit or those who did not receive any previous dental treatment at KAUFD were excluded. The legal guardians or parents of those who met the inclusion criteria were approached, and the research aim was introduced. Consent and assent forms were signed by those who agreed to participate.

**Figure 1:**
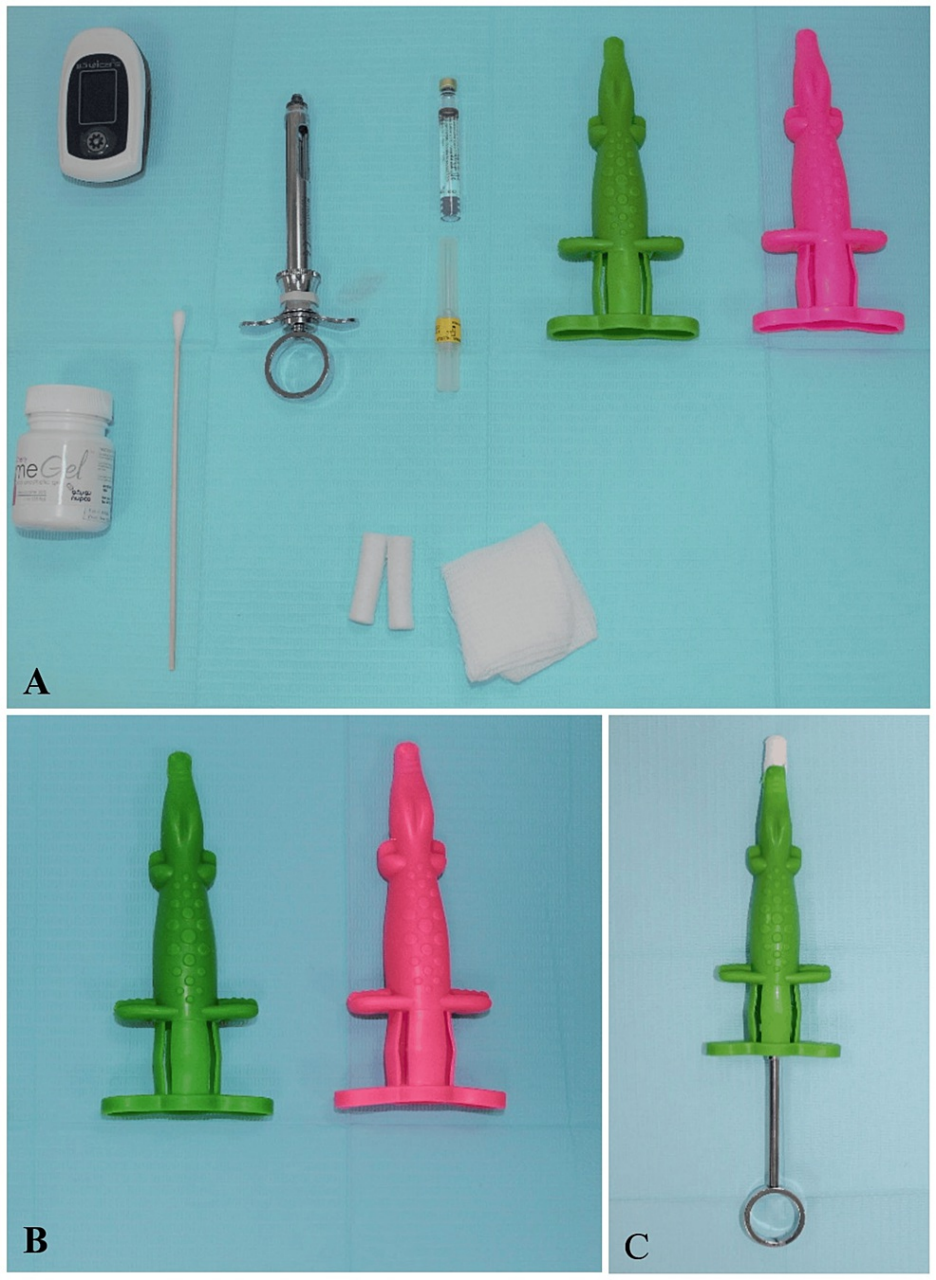
(A) Armamentarium used in the study; (B) the camouflaged dental syringe (Angelus™), which consists of an alligator-shaped syringe sleeve that covers the dental syringe; (C) the mouth of the alligator sleeve covers the local anesthesia needle, the trunk covers the syringe barrel of the traditional dental syringe, and the rear feet hold the finger grip.

Before the treatment, subjects were placed into a test or control group following a predetermined randomization sequence using number generator software. Participants would be assigned to either a camouflaged or control group according to the order in which they joined the study. In the control group, subjects received the BIA using a traditional dental syringe. In contrast, subjects in the test group received BIA with the camouflaged dental syringe. The camouflaged dental syringe (Angelus ™) used for the test group was an alligator-shaped syringe sleeve that covered the dental syringe and made it seem friendly without affecting its function. The alligator-shaped syringe sleeve has three main components: mouth, trunk, and rear feet. The mouth of the alligator sleeve covers the LA needle, the trunk covers the syringe barrel, and the rear feet hold the finger grip. The operator has to place his finger over the rear feet to gain support and then place his thumb inside the thumb ring of the dental syringe to introduce the solution of the LA (Figure 1).

A single-calibrated general dentist gave all the injections. All subjects were informed that the dentist would provide them with sleeping juice to help the teeth fall asleep, and the operator in both groups kept the needle part of the dental syringe covered with a cotton roll and out of the subjects’ sight. At the injection site, the oral mucosa was dried, and a topical Benzocaine Gel 20% (Sky-Caine Gel, Skydent, Calicut, India) was applied for two minutes. Then, a 30-gauge short needle (Septoject XL, Septodont, Saint-Maur-des-Fossés, France) was inserted in the mucobuccal fold without touching the bone. The LA agent Mepivacaine 2% with 1:100,000 epinephrine (Scandicaine 2% speciale, Septodont) was administered slowly. Finally, the needle was withdrawn. Due to the nature of the study, neither the operator nor the subjects were blinded to the treatment group.

**Figure 2:**
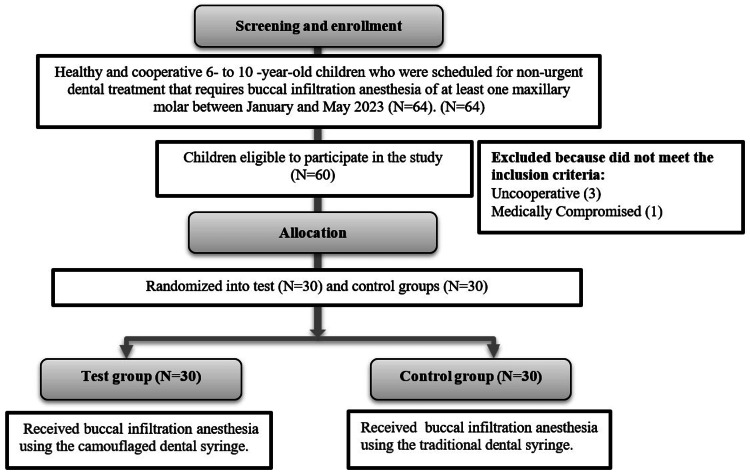
Study flow chart.

Using a pulse oximeter (iCare Fingertip Pulse Oximeter, blood oxygen and heartbeat measurement oximeter, OLED screen, White-At101C), patients’ heart rate (HR) was monitored and recorded as beats per minute (bpm) at three time points: as a baseline before LA administration, during the needle insertion, and one minute after LA administration was completed. In addition, during the administration of the LA, the subjects were recorded using a high-resolution camera. Later, two trained and calibrated investigators watched the videos and evaluated the anxiety level of the subjects using Venham’s Anxiety Rating Scale (VARS) [11] and the behavioral pain level using the Face, Leg, Activity, Cry, and Consolability Scale (FLACC) [12]. The VARS scores range from zero to five, where zero represents a relaxed, quiet child and five represents a screaming child. The FLACC is a valid, reliable, and commonly used scale that measures behavioral pain in children. The examiners observe the face, leg, activity, cry, and consolability of the subject during the dental procedure and assign a score of 0, 1, or 2 for each of the five criteria. The total scores range from zero to 10, with zero being relaxed and comfortable and 10 being severe discomfort, pain, or both [12]. The flow chart of the study is presented in Figure 2.

The inter-examiner reliability of the two investigators was evaluated using the intraclass correlation coefficient and was found to be excellent (ICC=0.976 for the VARS and ICC=0.985 for the FLACC). The statistician was blinded, and to ensure a balanced distribution, demographics and treatment-related factors were compared between the camouflaged and traditional dental syringe groups using the independent sample t-test and chi-square test. The HR data exhibited a normal distribution and were subjected to comparison across groups using an independent sample t-test. The VARS and FALCC were treated as ordinal data and were subjected to comparison between groups using the Mann-Whitney U test.

## Results

A total of 60 subjects, with a mean age of 8.3±1.3 years, were included in the study. Most of the treatments offered after the administration of the LA included the insertion of a stainless-steel crown (27, 45.0%). Fewer than half of the procedures (26, 43.3%) were performed on the right side. No statistically significant differences between the groups with respect to the demographic characteristics and parameters pertaining to treatment were observed. The demographic characteristics of the subjects included are presented in Table 1.

**Table 1:** The demographic characteristics of the subjects included (N=60). ^†^Independent sample t-test. ^‡^Chi-square test.

Variables	Total	Total N=60	Test group N=30	Control group N=30	P-value
Age	Mean ± SD	8.3 ± 1.3	8.4 ±1.4	8.2±1.3	0.505^†^
Sex	Male	30 (50.0)	14 (46.7)	16 (53.3)	0.606^‡^
	Female	30 (50.0)	16 (53.3)	14 (46.7)	
Provided treatment	Restoration	11 (18.3)	5 (16.7)	6 (20.0)	0.254^‡^
	Pulp treatment	3 (5.0)	0(0.0)	3 (10.0)	
	Stainless steel crown	27 (45.0)	16 (53.3)	11 (36.7)	
	Extraction	19 (31.7)	9 (30.0)	10 (33.3)	
Quadrant	Right	26 (43.3)	14 (46.7)	12 (40.0)	0.602^‡^
	Left	34 (56.7)	16 (53.3)	8 (60.0)	

**Figure 3:**
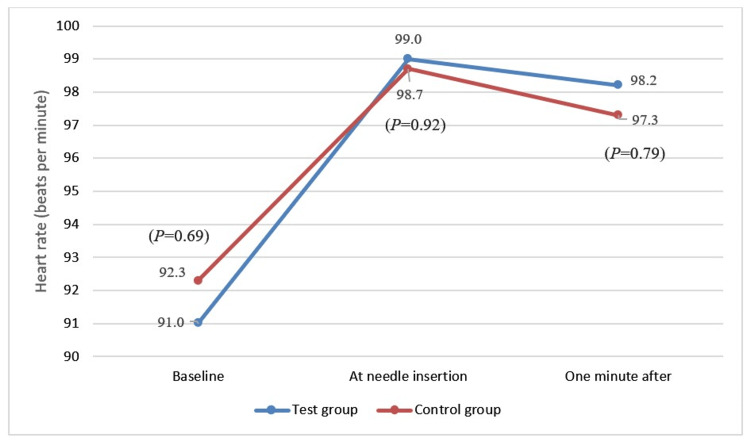
Comparison of the heart rate (beats per minute) between the group.

There were no statistically significant differences in HR observed between the groups at baseline, needle insertion, or one minute after the administration of LA. Regarding the VARS, most subjects in the test (22, 73.3%) and the control group (16, 53.3%) scored zero and were relaxed and quiet during the LA administration. Although the scores of the VARS in the subjects in the test group were somewhat lower compared to the subjects in the control group, the observed difference did not reach statistical significance (P=0.113). However, subjects in the test group showed significantly lower FLACC scores than the control group, and the difference was statistically significant (P=0.034). The results of the HR rate, FLACC, and VARS are shown in Table 2 and Figure 3.

**Table 2:** Comparison between the groups in mean heart rate (beats per minute), mean Face, Leg, Activity, Cry and Consolability Scale, and Venham’s Anxiety Rating Scale scores. ^†^Independent sample t-test. ^§^Independent sample Mann-Whitney U test. *Statistically significant (P<0.05). HR: heart rate, FLACC: Face, Leg, Activity, Cry and Consolability Scale, VARS: Venham Anxiety Rating Scale.

Variables		Test group N=30	Control group N=30	P-value
HR (beats per minute)	Baseline	91.0±12.8	92.3±13.2	0.693^†^
	At needle insertion	99.0±14.8	98.7±13.1	0.919^†^
	After one minute	98.2±13.3	97.3±13.5	0.788^†^
VARS	0	22 (73.3)	16 (53.3)	0.113^§^
	1	4 (13.3)	7 (23.3)	
	2	4 (13.3)	6 (20.0)	
	3	0 (0.0)	1 (3.3)	
	4	0 (0.0)	0 (0.0)	
	5	0 (0.0)	0 (0.0)	
FLACC	Mean ± SD	0.87±2.0	2.2±3.1	0.034*^§^

## Discussion

Dental anesthesia is considered one of the most common causes of fear and anxiety among children in dental clinics [3]. The sensory modalities, including visual stimuli, were found to affect the process of pain. Therefore, eliminating the visual input of the dental syringe during the administration of LA could reduce anxiety and improve behavior during LA administration [13].

The current study aimed to evaluate the effect of utilizing a camouflaged dental syringe in comparison to the traditional dental syringe during the administration of LA on the behavioral pain and anxiety levels among 6- to 10-year-old healthy and cooperative children. No statistically significant differences were observed between the groups concerning HR and VARS. Still, a significant decrease was observed in relation to the FLACC scale among the children who received LA with camouflaged dental syringes, indicating improved behavior.

In contrast to our findings, previous studies have reported that using a camouflaged syringe significantly reduced dental anxiety [8,14,15]. This can be attributed to the fact that, in the previous studies, only children with no prior experience with dental injection and anesthesia were included. In the current study, children with previous dental experience and cooperative behavior were included and, therefore, most probably anticipated receiving an injection. Also, their behavior during their previous dental visit(s) was reported in their records and measured based on the whole dental visit, not specifically on the dental injection. Further, studies to correlate the influence of the previous dental experience on the anxiety, pain, and behavior associated with camouflaged dental syringes are recommended.

The utilization of a camouflaged dental syringe was preferred by younger children (less than 10 years) and females [7,16]. In the current study, the age of subjects included was between 6 and 10, as this age group has good cognitive skills, ensuring that the utilization of a camouflaged dental syringe acts as a distractible tool for them and is highly preferred and favored [7,16].

Heart rate is a widely used and acceptable objective measure to detect anxiety levels [17]. There was a slight increase in the HR between baseline and at needle insertion in both groups. However, there were no statistically significant differences, but these increases were more frequent among the subjects in the camouflaged group compared to those in the traditional syringe group. This result is consistent with Melwani et al., who reported no significant differences between both groups in HR [7]. The findings are different from those reported by Padminee et al., which showed a significant increase in HR among the 6- to 11-year-old children who received their LA with a camouflaged syringe compared to the traditional syringe group [18].

Venham’s Anxiety Rating Scale is an objective measurement for evaluating a child’s anxiety. Although more subjects who received the LA using the camouflaged syringe were relaxed and quiet and scored zero (22, 73.3%) compared to 16 (53.3%) of the subjects who received the LA by the traditional syringe, the observed difference did not reach statistical significance (P=0.113). Ujaoney et al. and Monika et al. showed a significant reduction in the anxiety level with the use of a camouflaged syringe in children [14,15].

In the current study, the behavioral pain of children was evaluated using the FLACC scale. The findings demonstrated a statistically significant difference when using a camouflaged dental syringe. This is consistent with Monika et al., who reported better behavior when the camouflaged syringe was used [7,14,18].

One of the study’s limitations is that the only available camouflage syringe shape was a crocodile, which may not be every child’s favorite shape. The potential effectiveness of lowering anxiety in children could be enhanced by offering a variety of sizes and forms for camouflaged syringes, allowing children to make a choice. The inability to blind the operator to the type of syringe being used was another drawback. In addition, recruiting patients exclusively from a single institution may impede the generalizability of the study findings to a larger population. Conducting additional research across various locations has the potential to yield more generalizable results.

## Conclusions

Despite the limitations of the study, the effectiveness of employing a camouflaged dental syringe to hide the local anesthesia needle and syringe barrel from the sight of 6- to 10-year-old patients with prior dental experiences has been demonstrated in this study. This intervention has shown promise for enhancing the behavioral response to pain in this specific population. Therefore, camouflaged dental syringes may enhance the overall dental experience for pediatric dental patients and, as a result, can be suggested as an alternative to the conventional method of administering local anesthesia using dental syringes. Further research is warranted to assess the enduring impact of the camouflaged dental syringe on children's behavioral patterns and levels of anxiety, thereby enhancing our understanding of its efficacy.
